# 

**Published:** 1884-12

**Authors:** 


### INDEX TO VOLUME XLIX.

A. PAGE.

Abdominal Aorta, Aneurism of, Sears ... 22
A Collection of Curiosities, Editorial ... 419
Albuminuria Associated with the Habitual Use of Opium, Mann ... 492
Allen's Human Anatomy, Review ... 501
American Climatological Association ... 94
American Medical Association ... 156
Anatomical Bill, Editorial ... 38
Andrews, Edmund, Recent Cases in Surgical Practice ... 481
Aneurism of Abdominal Aorta, Sears ... 22
Aspirator Needle, Fatal Hæmorrhage from Use of, Jackson ... 385
As to the Value to Physicians of the Summer Vacation, Editorial ... 362
A Treatise on Diseases of the Eye, Review ... 370
Axford, W. L., Median Lithotomy ... 282

B.

Babcock, R. H., Physical Conditions Essential to the Production of Tympanitic Resonance ... 1
Bath, Continuous, Editorial ... 122
Berlin Polyclinic ... 155

PAGE.

Bettman, Boerne, Embedding Mass ... 103
Books and Pamphlets Received ... 95, 191, 575
Book Reviews ... 133, 263, 366, 424, 499
Botany of Illinois, Carter ... 115, 214
Bromwell, Byron, Heart and Thoracic Aorta, Review ... 366

C.

Calculus of Kidney, Orton ... 360
Cardiac Wounds, Roberts ... 33
Carter, J. M. G., Botany of Illinois ... 115, 214
Case of Aneurism of the Abdominal Aorta, Sears ... 22
Case of Fatal Chloroform Narcosis, Editorial ... 362
Cases of Insanity, Clevenger ... 105
Celloidin as an Embedding Mass, Bettman ... 103
Chicago and the Cholera, Editorial ... 126
Chicago Gynecological Society:
Meeting Oct. 31, 1884 ... 53
Meeting Nov. 21, 1884 ... 532
Chicago Medical Society:
Meeting May 5, 1884 ... 46
Meeting May 19, 1884 ... 267
Meeting June 16, 1884 ... 279
Meeting July 14, 1884 ... 435
Meeting Aug. 4, 1884 ... 439
Meeting Aug. 18, 1884 ... 446
Meeting Sept. 15, 1884 ... 567
Meeting Oct. 6, 1884 ... 570

PAGE.

Chicago Pathological Society:
Meeting Feb 11, 1884......... 458
Meeting Mar. 10, 1884......... 460
Meeting April 14, 1884......... 547
Meeting May 12, 1884......... 552
Meeting July 14, 1884......... 564
Chicago Society of Ophthalmology and Otology:
Meeting April 8, 1884......... 375
Meeting June 9, 1884......... 541
Meeting Aug. 12, 1884......... 543
Chloroform Anæsthesia, Roberts......... 29
Chloroform in Labor......... 526
Chloroform Narcosis, Fatal Case of......... 362
Cholera Abroad, Editorial...... 123
Cholera and Chicago, Editorial......... 126
Chronic Adhesive Perimetritis, Etheridge.........193, 321
Clevenger. S. V., Cases of Insanity......... 105
Clevenger, S. V., Letter......... 502
Continuous Bath, Editorial.... 122
Cook County Insane Asylum.. 502
Correspondence, Domestic...... 255, 470, 502
Correspondence, Foreign......... 513

D.

Danforth, I. N., Life Insurance......... 349
Davis, N. S., On Practice, Review......... 425
Deafness, Effect of Noises upon, Hawley......... 97
Die Diphtherie, Review......... 263
Diagnosis and Treatment of Diseases of the Ear......... 371
Diseases of the Heart and Thoracic Aorta, Review...... 366
Diseases of the Throat and Nose, Review......... 424
Dissection, Injection for Preserving Bodies, Freer......... 27

E.

Editorial...36, 122, 250, 362, 414, 496
Effect of Noises upon Certain Forms of Deafness, Hawley. 97
Eldridge, E. F., Separation of the Symphysis Pubis......... 495
Embedding Mass, Bettman..... 103
Ergot, Administration of......... 55

PAGE.

Etheridge, James H., Perimetritis.........193, 321
External Use of Chloroform in Labor, Svanberg......... 526

F.

Fatal Pelvic Hæmorrhage Following the Use of the Aspirator Needle, Jackson...... 385
Fenger, Chr., Excision of Hip and Knee Joints......... 289
Fenger, Chr., Surgical Treatment Pulmonary Gangrene.. 19
Field, The, of Disease, Review......... 428
Floating Hospital Association. 469
Fracture of the Humerus......... 46
Fractures, Vicious Union of, Roberts......... 34
Freer, O. T., Injections for Preserving Bodies......... 27

G.

German Medical Student and Physical Diagnosis, Editorial......... 414
Glycerine and Carbolic Acid for Preserving Bodies for Dissection, Freer......... 27
Gross, The S. D. Professorship, Correspondence......... 255
Gross, Sam'l D., Professorship, Editorial......... 250
Gross, Samuel David, In Memoriam......... 35
Gun-Shot Wounds of the Small Intestines, Parkes...59, 161

H.

Hæmorrhages, Fatality of Small, Roberts......... 30
Hamilton, F. H., Fractures and Dislocations, Review......... 500
Handbook of Vertebrate Dissection, Review......... 135
Hawley, Geo. H., Effect of Noises on Certain Forms of Deafness......... 97
Hebra's Continuous Tepid Water Bath, Editorial......... 122
Holmes, E. S., Muriate of Cocoaine......... 489
Hooper's Physician's Vade Mecum, Review......... 265

PAGE.

Holz, F. C., Our Children's Eyes and Books.................. 10

I.

Illinois State Board of Health: Meeting July 2, 1884........... 136
Illinois State Board of Health, Circular.............................. 157
Illinois, Medical Botany of, Carter.............................115, 214
Influence of the Mind on the Body, Review...................... 432
Injections during Labor......... 55
Injections of Glycerine and Carbolic Acid for the Preservation of Bodies for Dissection, Freer......... ............. 27
In Memoriam, Samuel David Gross.................................. 35
Insanity, Cases of, Clevenger.. 105
Introductory Lecture before the Classes of the Chicago Medical College, Sept. 23, 1884................................... 388
Iodoform, Use of During the Puerperium, Editorial......... 496

J.

Jackson, A. Reeves, Fatal Hemorrhage from Use of Aspirator Needle................ 85
Johnson, C. W., Translation.... 526

K.

Kidney, Calculus of, Orton..... 460

L.

Lagorio, A., Letter............470, 476
Lectures on the Principles and Practice of Medicine, Review................................. 425
Legal Medicine, Review......... 431
Letter from Boston... ............ 41
Letter from Darmstadt........... 513
Letter from Italy..............470, 476
Life Insurance and the Medical Profession, Danforth...... 349
Literary Notes...................... 422
Lydston, G. F., Puerperal Fever......................... ........ 46

M.

Malignant Tumors, Operative Delay in, Roberts............... 33

PAGE.

Mann, F. P., Use of Opium.... 492
Manual of Physiology, Review. 500
Marine Hospital Service, Changes............................. 286
Martin, H. Newell, Vertebrate Dissection, Review............. 135
Median Lithotomy, Axford.... 282
Medical Department Chicago Public Library .................. 467
Medical Jurisprudence and Toxicology, Review............ 499
Millard, H. B., Bright's Disease, Review...................... 433
Mackenzie, M., On the Throat, Review.............................. 424
More About Public Laundries, Editorial.................. 36
Muriate of Cocoaine, Holmes.. 489

N.

Nettleship, Edward, On the Eye, Review....................... 373
No Cure No Pay Decision........ 498

O.

Obstetrics, Practical Manual of, Review.......................... 133
Opium, Habitual Use of, Mann.................................. 492
Orton, Harlan N., Kidney Calculus................................ 360
Our Children's Eyes and Books, Holz........................ 10
Ovarian Cyst...... .................. 267
Owens, J. E., Introductory Lecture.............................. 388

P.

Parkes, Charles T., Gunshot Wounds of the Small Intestines..............................59, 161
Pericardial Wounds, Roberts.. 33
Perimetritis, Chronic Adhesive, Etheridge.............193, 321
Perineal Lacerations, Lydston 54
Perpetual Bath ..................... 507
Phlegmonous Inflammation, Roberts .......................... 52
Physical Conditions Essential to the Production of Tympanitic Resonance, Babcock 1
Politzer, Adam, On the Ear, Review ............................ 371

PAGE.

Pomeroy, Oren D., on the Ear, Review .......... 372
Post-Nasal Catarrh, Review... 264
Practical Manual of Obstetrics, Review .......... 133
Practical Treatise on Fractures and Dislocations, Review..... 500
Prince, David, Letter .......... 507
Puerperal Fever, Lydston...... 46
Puerperium, Iodoform in, Editorial .......... 496
Pulmonary Gangrene, Surgical Treatment of, Fenger .......... 19

R.

Recent Cases in Surgical Practice, Andrews .......... 481
Reese, John J., Medical Jurisprudence, Review .......... 499
Remarks on Excision of Hip and Knee Joints, Fenger..... 289
Richardson, B. W., Field of Disease, Review .......... 428
Roberts, F. T., Theory and Practice of Medicine, Review .......... 263
Roberts, John B., Surgical Delusions .......... 29
Rothe, Dr. Med., C. G., Die Diphtherie, Review .......... 263

S.

Sears, Mark H., Aneurism of the Abdominal Aorta .......... 22
Selections ..........59, 137, 526
Separation of the Symphysis Pubis During Labor, Eldridge .......... 495
Shall Medical Officers of the Army Engage in Private Practice? ..........253, 259
Small Intestines, Gunshot Wounds of, Parkes .......... 59
Society Proceedings .......... 47, 267, 375, 435, 529
State Medicine, Report of Committee .......... 275
Stevenson, Sarah Hackett, Letter .......... 513
Strangulated Hernia, Operative Delay in, Roberts .......... 29

PAGE.

Student's Guide to Diseases of the Eye, Review .......... 373
Styptics, Value of, Roberts ..... 30
Surgical Delusions, Roberts..... 29
Surgical Treatment of Acute, Circumscribed, Pulmonary Gangrene, Fenger .......... 19
Svanberg, A., Chloroform in Labor .......... 526
Symmetry of Normal Limbs, Roberts .......... 33
Synopsis of the Medical Botany of Illinois, Carter .......... 115
Tetanus, Traumatic, Necessary Fatality of, Roberts .......... 29

T.

Text Book on Diseases of the Ear, Review .......... 371
Theory and Practice of Medicine, Review .......... 263
Tidy, C. N., Legal Medicine, Review .......... .....
Treatise on Bright's Disease of the Kidneys, Review .......... 433
Trephining the Skull, Roberts 30
Tuke D. Hack, Influence of the Mind on the Body, Review .......... 432
Tympanitic Resonance, Physical Conditions Essential to the Production of, Babcock 1

U.

Uric Acid Calculus, Orton...... 360

V.

Verrier E., Manual of Obstetrics, Review .......... 133
Vertebrate Dissection, Review 135

W.

Wells, J. Soelberg, On the Eye, Review .......... 370
Woakes, Edward, Post-Nasal Catarrh, Review .......... 264

Y.

Yeo, G. F., Physiology, Review 500

### BOOKS RECEIVED.

BOOKS AND PAMPHLETS RECEIVED.

*A Manual of Obstetrics*, by A. F. A. King, M. D. Second edition. Henry C. Lea's Son & Co. Chicago: Jansen, McClurg & Co.

*Elements of Pharmacy, Materia Medica and Therapeutics*, by William White, M. D. Second edition. Henry Benshaw, London. Chicago: Jansen, McClurg & Co.

*Clinical Lectures on Mental Diseases*, by T. S. Clouston, M. D., with an abstract of the State and United States Laws relating to the custody of the insane, by C. F. Folsom, M. D. Henry C. Lea's Son & Co. Chicago: Jansen, McClurg & Co.

*Diseases of the Throat and Nose*, by Morel Mackenzie; vol. ii. P. Blakiston, Son & Co. Chicago: Jansen, McClurg & Co.

*Practical Pathology;* a Manual for Students and Practitioners, by G. Sims Woodhead, M. D. Henry C. Lea's Son & Co. Chicago: Jansen, McClurg & Co.

*Materia Medica and Therapeutics*, by J. Mitchell Bruce. Henry C. Lea's Son & Co. Chicago: Jansen, McClurg & Co.

*An Introduction to Pathology and Morbid Anatomy*, by T. Henry Green. Henry C. Lea's Son & Co. Chicago: Jansen, McClurg & Co.

*Post Nasal Catarrh and Diseases of the Nose causing Deaf-*

*ness*, by Edward Woakes, M. D. P. Blakiston, Son & Co. Chicago: W. T. Keener.

*The Formation of Poisons by Micro-Organisms; a Biological Study of the Germ Theory of Disease*, by G. V. Black, M. D., D. D. S. P. Blakiston, Son & Co. Chicago: W. T. Keener.

*Diseases of the Heart and Thoracic Aorta*, by Byron Bramwell, M. D., F. R. C. P. E. D. Appleton & Co. Chicago: Jansen, McClurg & Co.

*Die Zuckerharnruhe mit einer ausführlichen Diätetik für Zuckerkrakke Von Univ. Med;* Dr. Emerich Hertzka. Hans Feller, Carlsbad and Nice.

*The Medical Graduate and His Needs*, by George C. Wellner, M. D. Geo. S. Davis, Detroit.

*The National Dispensatory*, by Alfred Stille, M. D., L.L.D., and John M. Maisch, Phar. D. Henry C. Lea's Son & Co. Chicago: Jansen, McClurg & Co.

*Phthisis Pulmonalis—How affected by the climate of Colorado, and what are proper cases to be sent there.* L. H. Wood, M. D., Denver.

*Fifth Annual Report of the Illinois State Board of Health.* Springfield, 1883.

*Sixteenth Annual Report of the President of the Inebriates' Home.* Fort Hamilton, N. Y., 1883.

*The Proceedings of the Naval Medical Society.* Vol. ii, No. 1.

*Public Health Laws of Illinois, and Sanitary Memoranda.*

*Excision of the Rectum for Cancer: Operation.* Walter Coles, M. D.

*Medical Communications of the Massachusetts Medical Society.* Vol. xiii, No. 3. 1884.

